# Comparative Liability of Males and Females to Insanity

**Published:** 1851-01

**Authors:** 


### ARTICLE IV.

*On the comparative liability of Males and Females to Insanity, and their comparative curability when insane.* By EDWARD JARVIS, M. D., of Dorchester, Mass., pp. 32, 8 vo. (From the Author.)

This is an extremely interesting and valuable document. We regret that our limits will not permit us to quote largely from it; we must therefore content ourselves with a mere statement of the author's conclusions.

It would seem that males are more liable to insanity than females, although some countries are an exception to the rule; for instance Belgium and Norway. This is what we would naturally infer from the fact that men are more exposed to the exciting causes of the malady. Amongst males by far the most frequent cause of insanity seems to be intemperance, next to this is hereditary tendency: amongst women the most powerful cause is impaired health; and next to this ranks domestic trouble. Women also appear to be more frequently than men, affected with hereditary insanity.

From the reports of 189 Lunatic Hospitals, we find that amongst the males the recoveries amount to 40,6 *per cent.*, amongst the females to 43,9 *per cent.* Dr. Jarvis' explanation of this singular fact is doubtless as correct as it is philosophical. It amounts to about the following: That the causes which beget insanity in the male, are such as frequently produce structural lesions, whilst amongst females the preponderating causes are functional derangements. With this fact in view, we have no difficulty in reaching the third proposition in this pamphlet; that the rate of mortality of the insane, is much greater amongst males than females, the ratio of the latter to the former being as 15 to 21.

From pretty extensive research, Dr. Jarvis concludes that the ratio of mortality from nervous diseases is much higher among males than females, including both sane and insane. Of 2,169,875 deaths, of which 1,163,198 were males, and 1,066,677 females, 16,15 *per cent* of the males, and only 13, 85 *per cent* of the females died of nervous disorders. This little piece of statistics will doubtless astonish those doctors who are always assuring the ladies that their complaints are "all on the nerves;" or, supposing that women are really more liable than men, to nervous disorders, it may serve to explain the origin of the old saying, that "a sickly woman never dies."

